# *Helicobacter pylori*‐negative extra‐nodal marginal zone B‐cell lymphoma of Mucosa‐Associated Lymphoid Tissue (MALT) type following Roux‐en‐Y Gastric Bypass (RYGB)

**DOI:** 10.1002/ccr3.5261

**Published:** 2022-01-23

**Authors:** Zachary R. Eagle, Francis Essien, Kimberly Zibert, Charles Miller, Melissa Van Dellen, Rina Eden, Ross Pinson

**Affiliations:** ^1^ Department of Internal Medicine Keesler Medical Center Keesler Air Force Base Keesler AFB Mississippi USA; ^2^ Division of Gastroenterology Department of Internal Medicine San Antonio Medical Center San Antonio Texas USA; ^3^ Division of Pathology Department of Internal Medicine Keesler Medical Center Keesler Air Force Base Keesler AFB Mississippi USA; ^4^ Division of Gastroenterology Department of Internal Medicine Keesler Medical Center Keesler Air Force Base Keesler AFB Mississippi USA

**Keywords:** dysphagia, extra‐nodal marginal zone lymphoma, gastric pouch, gastric remnant, *Helicobacter Pylori* negative, MALT Lymphoma, Roux‐en‐Y gastric bypass

## Abstract

Gastric MALT lymphoma is a common type of non‐Hodgkin's lymphoma that has the potential for cure in patients found to have concomitant *Helicobacter pylori* (*H. pylori*) infection. This case report explores the evaluation, diagnosis, and treatment of *H. pylori*‐negative MALT lymphoma in a patient with a history of a RYGB.

## Graphical abstract

In patients with gastric MALT lymphoma and a RYGB, regardless of *H. pylori* status, surveillance of both the gastric pouch and remnant should be performed as it can occur in both locations despite anatomical separation.

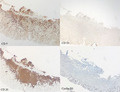

## 1 INTRODUCTION

Extra‐nodal marginal zone lymphoma (MZL) of MALT type compose 7% of all non‐Hodgkin's lymphomas.^1^ Approximately one‐third present as a primary gastric lymphoma; 90% are associated with *Helicobacter pylori* (*H. pylori*).^1^, ^2^ The etiology of *H. pylori*‐negative lymphoma of MALT type remains controversial. Differentiating *H. pylori* negative from *H. pylori* lymphoma of MALT type is important in regard to treatment and prognosis. The incidence of primary MALT lymphoma of the gastric remnant or gastric pouch is not well defined but appears to be quite rare with less than 35 cases reported worldwide.^3^, ^4^, ^5^, ^6^ In this case report, we discuss the suspected etiologies, diagnosis, treatment, and outcome of a 36‐year‐old female patient found to have *H. pylori*‐negative gastric lymphoma of MALT type. The case is further complicated by history of Roux‐en‐Y gastric bypass (RYGB) for treatment of refractory gastroesophageal reflux disease (GERD).

## 2 CASE DESCRIPTION

**FIGURE 1:**
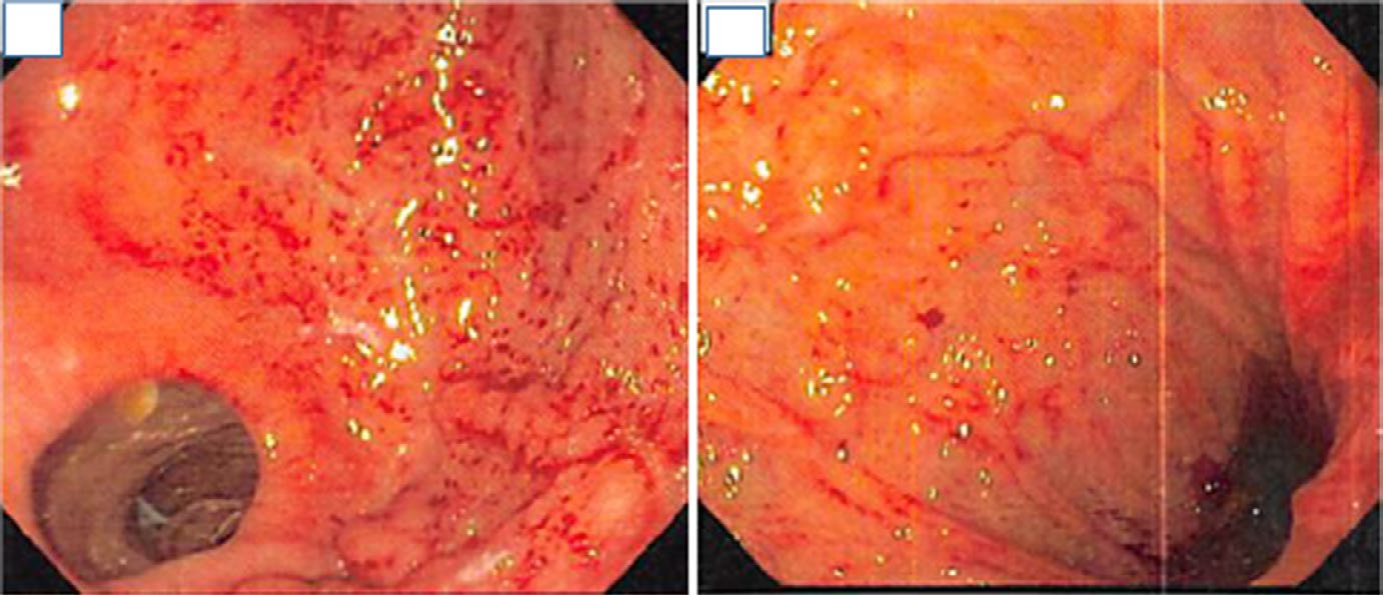
Diffuse, punctate erythema, edema and friability in the gastric pouch (Left) and, to a lesser degree, in the gastric body of the excluded stomach (Right)

**FIGURE 2:**
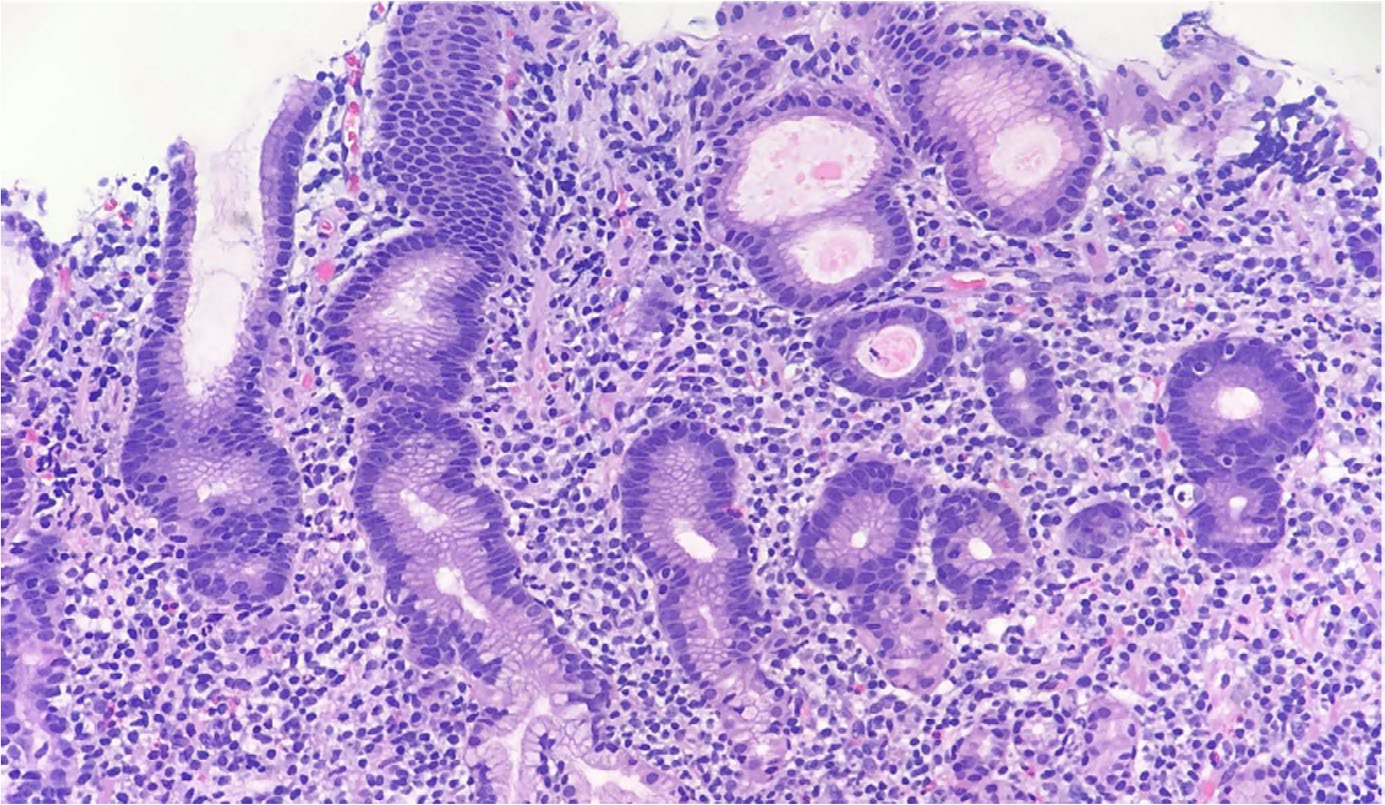
Gastric mucosal biopsy, H&E, 200×: dense infiltration of small to medium‐sized monocytoid lymphocytes and characteristic lymphoepithelial lesions

**FIGURE 3:**
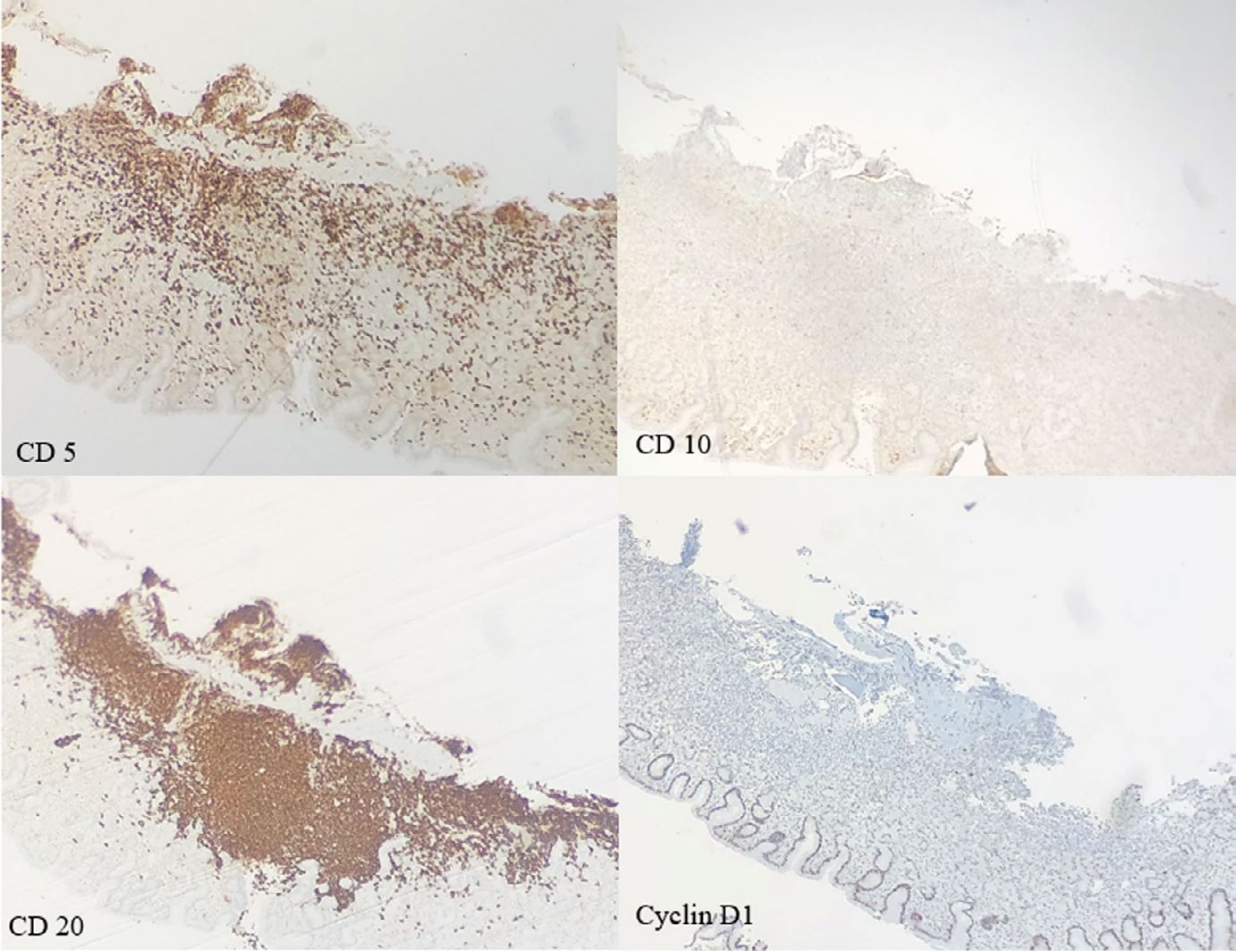
(40×) Focally, there is an atypical lymphoid infiltrate composed primarily of enlarged, mature lymphocytes with "monocytoid" features and associated immunoblastic and centroblastic lymphocytes. A predominance of CD5 and CD20 staining indicates B‐lymphocyte predominance in the atypical infiltrate. Staining for CD10 and Cyclin D1 within the lymphoid infiltrate was largely unremarkable

A 36‐year‐old African American female patient with a history of esophageal dysmotility secondary to systemic sclerosis and refractory GERD status post‐RYGB presented to her primary care provider with complaints of daily nausea, non‐bloody emesis, dysphagia, and abdominal pain refractory to all medical therapy. The patient had undergone endoscopic evaluation multiple times for this complaint which were initially limited to the gastric pouch secondary to surgical anatomy with no abnormalities noted. Laboratory analysis was notable for normal CBC, normal comprehensive metabolic panel CMP, and negative serologic and stool Helicobacter pylori antigen testing. The patient was referred back to gastroenterology for repeat upper endoscopic evaluation. The gastric pouch was notable for diffuse edema, punctate erythema, and friability (Figure 1). Biopsies demonstrated chronic gastritis with atypical lymphoid infiltrate that was found to have high CD20 and CD 5 expression. Further staining with CD10 and Cyclin D1 was unremarkable; however, testing demonstrated strong expression of BCL‐2 (cytoplasmic pattern) and a significant predominance of kappa staining within the infiltrate supporting the diagnosis of MALT lymphoma (Figures 2 and 3). A single balloon enteroscopy was then performed for evaluation with biopsies obtained of the gastric remnant. Similar gross findings were seen in the gastric remnant; however, biopsies showed dense lymphoid infiltrate consistent with MALT lymphoma. All biopsies were negative for *H. pylori* by immunohistochemical stains. Biopsies performed a year prior to her current presentation at the time of her RYGB surgery were reviewed and confirmed to be negative for H pylori infection as were serologic and stool antigen tests.

After further evaluation with positron emission tomography and computed tomography (PET‐CT), the patient was diagnosed with H pylori‐negative gastric MALT lymphoma, Lugano stage I, and Ann Arbor IE. Endoscopic ultrasound was not used in the staging of this patient due to the complexity of her post‐surgical anatomy. After discussion with the patient, the decision was made to treat with rituximab given the risk of large field radiation and her underlying systemic sclerosis. Repeat endoscopy with biopsy after 4 weeks of treatment showed no appreciable gross or histologic changes. Having failed immunosuppressant therapy, patient was initiated on radiation therapy for a total dose of 30 Gy. On follow‐up, symptoms had improved; repeat single balloon enteroscopy showed mucosal improvement. Biopsies were notable for focal atypical lymphoid infiltrate with monocytoid cytomorphology and focal lymphoepithelial lesion formation, compatible with focal, residual marginal zone lymphoma (partial histologic regression). The amount of atypical lymphoid infiltrate was too small for further assessment by immunohistochemistry. Repeat endoscopy was planned within the next three months but unfortunately with the advent of COVID patient was lost to follow‐up.

## 3 DISCUSSION

Development of gastric MALT lymphoma appears to arise from two distinct pathways both involving dysregulation of nuclear factor kappa light chain enhancer of activated B‐cell (NF‐kB) activity.^7^ There is a B‐cell receptor (BCR)‐dependent NF‐kB activation pathway and BCR‐independent NF‐kB activation pathway.^7^, ^8^ BCR‐dependent NF‐kB activation relies on persistent antigen stimulation which elicits inflammation and accelerated lymphoid proliferation through a polyclonal B‐cell response.^7^, ^9^, ^10^ This pathway is also recognized as an anti‐microbial responsive lymphoid proliferation as when the stimulating antigen is removed, the inflammation and lymphoid proliferation resolves.^11^ The above mechanism is further supported by the effectiveness of eradication therapy in the treatment of *H. pylori*‐associated MALT lymphoma. Current literature suggests regression in up to 83% of *H. pylori*‐associated MALT lymphoma cases when given triple or quadruple therapy.^12^, ^13^ Previous studies in patients that have undergone a RYGB have shown an increase in cell proliferation as well as an associated downregulation in cell apoptosis.^14^ This alteration in cell turnover seems unique to patients that have undergone previous RYGB and may further contribute to an increase in the presence of malignant lesions over time. However, research following cancer incidence in patients that previously underwent a RYGB vs patients that opted for non‐surgical management showed an overall decrease in cancer incidence over time, likely related to the effects of weight loss.^15^

Though uncommon, there are reports showing *H. pylori*‐negative MALT lymphoma similar to our case presented above. The predominating theory suggests a BCR‐independent NF‐kB activation pathway. Though there is a clear theory describing the pathway for lymphoid proliferation in *H. pylori*‐negative patients, the exact mechanism is yet to be determined and is likely multifactorial. One known etiology is a pseudo‐negative *H. pylori*‐associated MALT lymphoma. In these patients, *H. pylori* testing is negative due to previous use of antibiotics, bismuth, proton‐pump inhibitors (PPIs), or a combination of the three despite active infection with *H. pylori*.^16^ Some studies indicate that certain chromosomal translocations or tumor suppression gene mutations can cause constitutive lymphoid proliferation independent of a stimulating antigen.^17^ Multiple publications demonstrate a high incidence of translocation (11;18)(Q21;Q21) in *H. pylori*‐negative MALT lymphomas.^7^, ^18^, ^19^, ^20^ These translocations cause a fusion of the N‐terminus of the API2 gene to the C‐terminus of the MALT1 gene and generate a functional API2–MALT1 fusion product, which can constitutively activate the NF‐kB pathway.^21^

Presence of the t(11;18)(q21:q21) is seen in up to 30% of all gastric MALT lymphomas and is demonstrated in up to 68% that are at stage IIE or above.^22^ Unfortunately, the current available data have not closely evaluated the incidence of these translocations in patients with *H. pylori*‐negative gastric MALT lymphoma, though there are multiple studies that show expression of these translocations in up to 88% of these patients.^7^, ^18^, ^19^, ^20^, ^21^, ^22^ Regardless, the presence of t(11;18)(q21:q21) can help guide therapy as they are only seen in approximately 3% of gastric MALT lymphomas that do respond to traditional *H. pylori* eradication therapy.^22^ Further studies noted that the presence of t(11;18)(q21:q21) predicted poor response to alkylating agents (chlorambucil or cyclophosphamide) but was unable to predict the response to rituximab.^23^, ^24^

Treatment of patients with *H. pylori*‐negative MALT lymphoma is complicated and differs depending upon the patient's comorbidities, staging, and the presence or absence of translocations. Current literature suggests using radiation therapy for patients with early stage (Lugano I/II) gastric MALT lymphoma without evidence of *H. pylori* infection and reported clinical remission rates in up to 100% of patients.^25^, ^26^ If radiation therapy fails or the patient is found to be at an advanced stage, treatment with immunotherapy (Rituxumab) can be trialed and has shown complete response in up to 46% of patients.^24^ Prior to the discovery of *H. pylori*, targeted gastric resection was used to great therapeutic effect and long‐term survival.^24^, ^27^, ^28^, ^29^ However, more recent studies suggest that organ‐conserving therapy presents no long‐term disadvantages but spares the patient from permanent nutritional and metabolic derangements.^28^, ^29^ For these reasons, surgical treatment of gastric MALT lymphoma is rarely pursued. Interestingly, in patients that have previously undergone a RYGB that are found to have isolated MALT lymphoma of the gastric remnant, surgery is an option for definitive therapy as there is already a significant pre‐existent risk for nutritional/metabolic derangements given the previous gastric bypass.^3^, ^4^, ^24^, ^27^ However, in the above patient, the MALT lymphoma was present in both the gastric remnant and pouch, as such, surgery was not considered as an available option.

Though chromosomal changes are not diagnostic, in the case above, karyotype analysis for t(11;18)(q21:q21) was negative. Additionally, other commonly associated chromosomal abnormalities to include t(14;18)(q32;21), t(1;14)(p22;q32), t(3;14)(p13;q32), and trisomy 3 were unremarkable. Rituximab was preferred over radiation therapy in our patient with history of systemic sclerosis but was ineffective. She has shown endoscopic and histologic improvement with radiation therapy with 3‐month repeat follow‐up endoscopy pending.

In conclusion, *H. pylori*‐negative gastric lymphoma of MALT type is an uncommon presentation of non‐Hodgkin lymphoma. Though the mechanism appears well researched, the specific etiology remains controversial. In patients with gastric lymphoma of MALT type, it is important to rule out *H. pylori* infection. If negative, further evaluation of the t(11;18)(q21:q21) can help further guide therapy and predict patient outcomes. Lastly, MALT lymphoma in patients that have undergone Roux‐en‐Y gastric bypass is uncommon with less than 40 cases in the reported literature. Thus, diagnosis can be delayed due to mimicking symptoms typically attributed to the bypass. Evaluation, not only of the gastric pouch, but also the gastric remnant should be performed in all at risk patients as gastric MALT lymphoma can occur in both locations despite post‐operative anatomical separation.

## CONFLICT OF INTEREST

There are no conflicts of interests to report, and no funding was provided for this research.

## AUTHOR CONTRIBUTIONS

Zachary R Eagle MD^1^, Francis Essien DO^1^, Kimberly Zibert DO^2^, Charles Miller MD^2^, Melissa Van Dellen MD^3^, Rina Eden DO^3^, and Ross Pinson MD^1,4^ involved in study conception and design. Zachary R Eagle MD^1^, Melissa Van Dellen MD^3^, Rina Eden DO^3^, and Ross Pinson MD^1,4^ involved in data collection. Zachary R Eagle MD^1^, Kimberly Zibert DO^2^, Charles Miller MD^2^, Melissa Van Dellen MD^3^, Rina Eden DO^3^, and Ross Pinson MD^1,4^ involved in analysis and interpretation of results. Zachary R Eagle MD^1^, Francis Essien DO^1^, and Ross Pinson MD^1,4^ involved in draft manuscript preparation. All authors discussed the results and contributed to the final manuscript.

## CONSENT

Written informed consent was obtained from the patient to publish this report in accordance with the journal's patient consent policy.

## Data Availability

Data sharing is not applicable to this article as no new data were created or analyzed in this study.
